# Combination chemotherapy plus dasatinib leads to comparable overall survival and relapse‐free survival rates as allogeneic hematopoietic stem cell transplantation in Philadelphia positive acute lymphoblastic leukemia

**DOI:** 10.1002/cam4.2153

**Published:** 2019-04-23

**Authors:** Jeremy Chang, Dan Douer, Ibrahim Aldoss, Golnaz Vahdani, Ah‐Reum Jeong, Zunera Ghaznavi, Sherry Zhang, George Yaghmour, Kum‐Ja Lee, Ashley Weissman, Mojtaba Akhtari

**Affiliations:** ^1^ Los Angeles County ‐ University of Southern California Los Angeles California; ^2^ Jane Anne Nohl Division of Hematology and Center for the Study of Blood Diseases Norris Cancer Center Los Angeles California; ^3^ City of Hope Duarte California; ^4^ Harbor‐UCLA Medical Center Torrance California; ^5^ University of Southern California School of Pharmacy Los Angeles California

**Keywords:** acute lymphoblastic leukemia, Dasatinib, Philadelphia chromosome, stem cell transplantation, survival

## Abstract

**Background:**

The Philadelphia chromosome is associated with a poor prognosis in acute lymphoblastic leukemia (ALL). While hematopoietic stem cell transplantation (HSCT) has been regarded as a favorable treatment option in adult Philadelphia‐positive (Ph+) ALL, its benefit is less clear in the era of newer generation tyrosine kinase inhibitors (TKIs) like dasatinib.

**Methods:**

This was a retrospective study that analyzed the outcomes of adult patients with Ph+ ALL treated with either combination chemotherapy plus dasatinib or combination chemotherapy plus dasatinib followed by allogeneic HSCT.

**Results:**

A total of 70 patients were included; 30 (42.9%) underwent allogeneic HSCT while 40 (57.1%) received only chemotherapy plus dasatinib. In comparing overall survival (OS) rates, results between the 2 groups were similar with a 1‐year OS of 93.3% versus 100% (*P* = 0.20), 2‐year OS of 89.8% versus 86.2% (*P* = 0.72), and 3‐year OS of 76% versus 71.3% (*P* = 0.56) in the transplant versus nontransplant groups, respectively. The 3‐year relapse‐free survival (RFS) rates were also similar at 70.5% in the transplant group and 80.1% in the nontransplant group (*P* = 0.94). Subgroup analyses were performed for patients with specific poor prognostic factors (higher white blood count, older age, positive minimal residual disease status), but results again showed no significant survival difference between transplant and nontransplant patients.

**Conclusions:**

While HSCT has historically led to a survival advantage in Ph+ ALL, the results of our study demonstrate that it may have a less beneficial role in the era of newer generation TKIs such as dasatinib.

## 1 BACKGROUND

Acute lymphoblastic leukemia (ALL) is a hematological malignancy characterized by the proliferation of immature lymphocytes in the bone marrow, peripheral blood, and extramedullary sites. Chromosomal and molecular abnormalities divide ALL patients into multiple subtypes and provide prognostic information that impacts management decisions. The presence of the Philadelphia chromosome is one such abnormality that is associated with a poor prognosis in all patients with ALL. The large‐scale Eastern Cooperative Oncology Group (ECOG) 2993 trial looking at over 1500 patients with ALL previously demonstrated that the 5‐year overall survival (OS) rate among adult patients with Philadelphia‐positive (Ph+) ALL was 25% compared to 41% in Philadelphia‐negative (Ph‐) ALL.^1^ Subsequent karyotype analysis of this trial confirmed the negative prognostic impact of Ph+ status with a significantly decreased event free survival (EFS) rate of 16% in Ph+ ALL patients compared to 36% in Ph‐ ALL as well as a re‐confirmation of the decreased 5‐year OS (22% vs 41%).^2^ The frequency of Ph+ ALL increases in older populations, with 25% of patients 40‐49 years and up to 40% of patients >50 years in the Ph + subtype.^2^, ^3^

Before the emergence of tyrosine kinase inhibitor (TKI) therapy or hematopoietic stem cell transplantation (HSCT), the 3‐year OS rates for adults with Ph+ ALL treated with chemotherapy alone were estimated to be less than 20%.^4^ The subsequent use of allogeneic HSCT following first complete remission (CR1) led to an improvement in survival in this population with 3‐year OS rates ranging from 36% to 44% in multiple studies.^5^, ^6^ The ECOG 2993 trial again confirmed the survival benefit of HSCT in the pre‐TKI era by demonstrating 5‐year OS rates of 44%, 36%, and 19% in patients who underwent matched related donor HSCT, matched unrelated donor HSCT, and chemotherapy alone, respectively.^7^ As a result, allogeneic HSCT was aggressively pursued in Ph+ ALL patients during period.

The development of TKIs for the management of Ph+ hematological malignancies represented a monumental advancement in cancer therapy. The combination of chemotherapy with imatinib, a first‐generation TKI, in Ph+ ALL patients led to significantly higher rates of CR and OS in several studies when compared to prior treatment regimens.^4^, ^8^, ^9^ In the pediatric population, the use of imatinib with chemotherapy also led to comparable outcomes as allogeneic HSCT, particularly in standard risk patients; transplant is, therefore, no longer a standard recommended therapy for the majority pediatric Ph+ ALL patients in CR1.^10^, ^11^ Likewise, this advancement has made the benefit of HSCT less clear in the adult Ph+ ALL population, with some studies showing no statistically significant difference in 3‐year OS or EFS between patients who received only chemotherapy plus imatinib versus those who received chemotherapy plus imatinib followed by HSCT.^12^, ^13^

Compared to the first‐generation TKIs, dasatinib is a second‐generation TKI that inhibits a broader range of kinases including those of the SYK family (spleen tyrosine kinase and ZAP‐70) and SRC family, with the latter serving as an alternative signaling pathway in imatinib‐resistant ALL.^14^ Other previously demonstrated benefits of dasatinib include increased potency in inhibiting the in vitro growth of cells with wild‐type BCR‐ABL compared to imatinib as well as its penetration of the blood brain barrier for activity against central nervous system (CNS) leukemia.^14^, ^15^ Though there are no studies directly comparing outcomes from dasatinib versus imatinib, previous phase II, and phase III studies reported increased activity from dasatinib in patients with relapsed or refractory Ph+ ALL as well as those who were either unable to tolerate or had disease resistant to imatinib.^16^, ^17^, ^18^

With the development of more potent, newer generation TKIs such as dasatinib, the role of HSCT in the management of adult Ph+ ALL patients has become even less clear. Our study describes data regarding the use of dasatinib in combination with chemotherapy in adult patients with Ph+ ALL treated in a real life setting.

## 2 PATIENTS AND METHODS

### 2.1 Patient population

This was a retrospective cohort study conducted at the Los Angeles County Medical Center and Norris Comprehensive Cancer Center, both affiliated with the University of Southern California (USC), to analyze the outcomes of adult patients aged >18 years and <70 years who were diagnosed with Ph+ ALL between 2005 and 2018. The study was approved by the institutional review board (IRB) at the Norris Comprehensive Cancer Center. The patients were grouped into 2 cohorts for analysis: those who were treated with only combination chemotherapy plus dasatinib and those who were treated with combination chemotherapy plus dasatinib followed by allogeneic HSCT. Patients had to achieve morphological CR and survive induction therapy in order to be eligible for analysis. Allogeneic HSCT was the preferred method of treatment for all patients in this study; requirements for undergoing transplant included an available donor, insurance coverage, and the presence of a caregiver at home. All transplant patients underwent allogeneic HSCT while in CR1. Minimal residual disease (MRD), defined as the presence of leukemic cells below the threshold of detection by morphologic methods, was assessed with real‐time quantitative polymerase chain reaction (qRT‐PCR) assays for the BCR‐ABL1 fusion gene. In this study, MRD negative disease was defined as the absence of a BCR‐ABL1 transcript as determined by qRT‐PCR with a sensitivity of 0.01%. The electronic medical records at both medical centers contained all patient information including demographic data, laboratory and pathology reports, details of treatment, and outcomes.

### 2.2 Chemotherapy regimens

Chemotherapy regimens included the combination of cyclophosphamide, vincristine, doxorubicin, and dexamethasone (hyper‐CVAD), a variation in the Berlin‐Frankfurt‐Münster protocol (BFM‐like), or a pediatric‐inspired ALL regimen developed at the University of Southern California (USC ALL regimen) using pegaspargase.^19^ In the nontransplant group, those who tolerated chemotherapy and whose disease remained in remission received the maximum number of cycles of therapy. Patients in the transplant group received a varying number of cycles until a transplant donor was identified and underwent HSCT when their disease was in morphological CR1. Dasatinib was administered concurrently with all cycles of chemotherapy, starting at 140 mg/d with dose de‐escalation if patients experienced medication toxicity.

### 2.3 Transplant regimens

Prior to HSCT, all patients underwent evaluation with a complete blood count, liver function and renal testing, bone marrow biopsy, and cardiopulmonary function tests. Allogeneic HSCT was performed with matched related donors, matched unrelated donors, and haploidentical donors. Conditioning regimens included cyclophosphamide with total body irradiation (TBI), busulfan with cyclophosphamide, fludarabine with melphalan, and fludarabine with TBI. Prophylaxis for graft‐versus‐host disease (GVHD) was provided with combination tacrolimus and methotrexate, tacrolimus and mycophenolate mofetil (MMF), cyclosporine and methotrexate, or cyclosporine and MMF. Allogeneic stem cells were obtained from bone marrow or peripheral blood and were infused intravenously on day 0 through a central venous catheter. Granulocyte colony‐stimulating factor (G‐CSF) was started on day +7 and continued until neutrophil engraftment. Following transplant, all patients received anti‐viral prophylaxis with acyclovir, anti‐fungal prophylaxis with either an azole or echinocandin, and anti‐bacterial prophylaxis with levofloxacin if the absolute neutrophil count (ANC) was <500 cell per mm^3^. Dasatinib was subsequently initiated approximately 100 days post‐HSCT; different doses of dasatinib were used based on the patients’ blood counts and tolerance, and therapy was continued for 2 years. Bone marrow biopsies were performed 100 days after transplant to assess for disease response to therapy.

### 2.4 Statistical analysis

For this study, a landmark survival analysis was performed with several prespecified landmark time points. OS was defined as the time from diagnosis of Ph+ ALL until patient death. Relapse‐free survival (RFS) was defined as the time to reappearance of blasts in the blood or BM (>5%) or in any extramedullary site following CR1. Statistical analysis was performed using the Mann‐Whitney U Test and Fisher's Exact Probability Test with two‐tailed *P*‐values <0.05 deemed significant.

## 3 RESULTS

A total of 70 adult Ph+ ALL patients were eligible for analysis in this study. Forty‐five patients (64.3%) were male and the median age at the time of diagnosis was 44 years (range 21‐69). The median follow‐up time was 15 months (range 1‐131). The characteristics of patients were similar and are listed in Table 1.

**Table 1:** Patient characteristics

Characteristics	Transplant number (%)	Nontransplant number (%)	*P*‐value^a^
Number of patients	30	40	
Age (y)			0.97
Median	45	44	
Range	21‐65	21‐69	
Age group (y)			0.85
<35	9 (30)	11 (27.5)	
35‐60	20 (66.7)	26 (65)	
>60	1 (3.3)	3 (7.5)	
Sex			0.46
Male	21 (70)	24 (60)	
Female	9 (30)	16 (40)	
Ethnicity			0.90
White	7 (23.3)	8 (20)	
Hispanic	22 (73.3)	30 (75)	
Other	1 (3.3)	2 (5)	
Cytogenetics/FISH			0.33
Monosomy chromosome 7	0 (0)	2 (5)	
T‐cell ALL	1 (3.3)	0 (0)	
MLL gene rearrangement	1 (3.3)	0 (0)	
CNS involvement at diagnosis			0.50
Yes	0 (0)	2 (5)	
No	30 (100)	38 (95)	
Type of insurance			**0.03**
Private	17 (56.7)	11 (27.5)	
Public	13 (43.3)	29 (72.5)	
Comorbidities			0.71
Hypertension	3 (10)	12 (30)	
Type 2 diabetes mellitus	3 (10)	11 (27.5)	
Solid malignancy	1 (3.3)	2 (5)	
Congestive heart failure	1 (3.3)	1 (2.5)	
Chronic kidney disease	1 (3.3)	1 (2.5)	
Diagnosis to transplant (y)			
<1	28 (93.3)	—	
>1	2 (6.7)	—	
Fusion genes			0.80
p190	19 (63.3)	27 (67.5)	
p210	11 (36.7)	13 (32.5)	

Abbreviations: ALL, acute lymphoblastic leukemia; CNS, central nervous system; FISH, fluorescence in situ hybridization; MLL, mixed‐lineage leukemia.

a Bold type indicates statistical significance.

For chemotherapy regimens in both the transplant and nontransplant groups, the USC ALL regimen was used in 47 (67.1%) patients, hyper‐CVAD in 20 (28.6%), and the BFM‐like protocol in 3 (4.3%) patients. While in morphological CR, 30 (42.9%) patients underwent allogeneic HSCT and all received subsequent posttransplant dasatinib therapy. Median time of dasatinib initiation following transplant was at day 102. Matched related donor transplants were performed in 14 (46.7%) patients, matched unrelated donor transplants in 7 (23.3%), and haploidentical donor transplants in 9 (30%) patients. The full details of transplantation are listed in Table 2. Forty (57.1%) patients did not undergo transplant and received only chemotherapy with dasatinib. Dasatinib was given concurrently with all cycles of chemotherapy and continued indefinitely as maintenance therapy in all patients of the nontransplant group until disease progression. No patients in the transplant group had CNS involvement from leukemia at the time of diagnosis and only 2 (5%) patients in the nontransplant were found to have CNS involvement.

**Table 2:** Transplantation characteristics

Characteristics	Transplant patients Number (%)
Number of patients	30
Type of transplant	
Matched related donor	14 (46.7)
Matched unrelated donor	7 (23.3)
Haploidentical donor	9 (30)
Conditioning regimen	
Cyclophosphamide with TBI	14 (46.7)
Busulfan with cyclophosphamide	6 (20)
Fludarabine with melphalan	6 (20)
Fludarabine with TBI	4 (13.3)
GVHD prophylaxis	
Tacrolimus and methotrexate	16 (53.3)
Tacrolimus and MMF	9 (30)
Cyclosporine and methotrexate	3 (10)
Cyclosporine and MMF	2 (6.7)
Posttransplant dasatinib initiation	
Median (d)	102
Range (d)	53‐140

Abbreviations: GVHD, graft‐versus‐host disease; MMF, mycophenolate mofetil; TBI, total brain irradiation.

### 3.1 Minimal residual disease

In the transplant group, 25 (83.3%) patients were able to undergo MRD analysis and 17 (68%) of these patients were found to be MRD positive following induction therapy. In the nontransplant group, 20 (50%) patients underwent MRD analysis and 13 (65%) of these patients were MRD positive after induction therapy. Patients were offered allogeneic HSCT regardless of MRD positive or negative status if a transplant donor was available and all other transplant criteria (insurance coverage and availability of a caregiver at home) were met.

**Figure 1:**
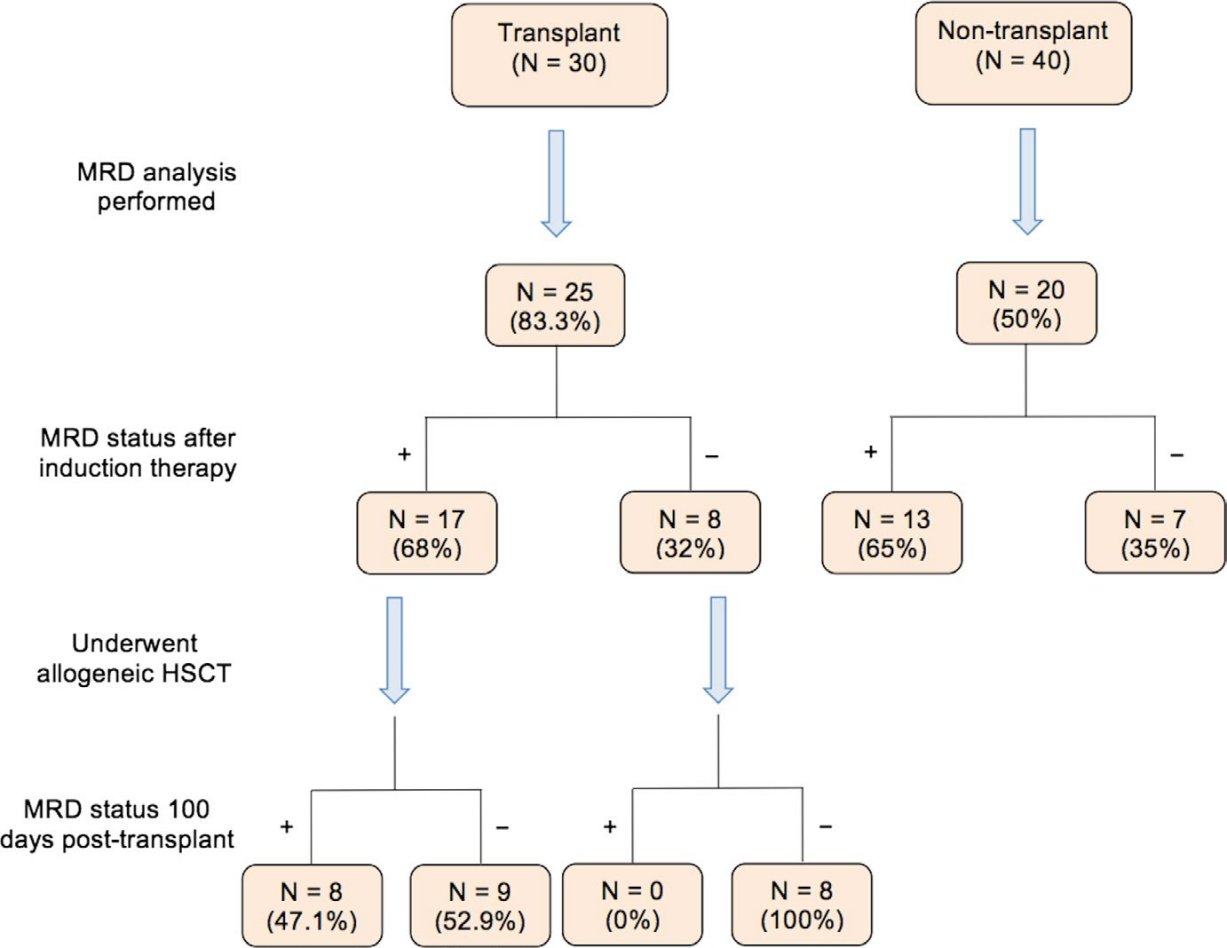
Minimal residual disease (MRD) status of transplant and nontransplant patients following induction therapy and at 100 days posttransplant

In the allogeneic HSCT group, bone marrow biopsies were performed 100 days after transplant to assess for disease response to therapy. Results revealed that of the 17 patients in the transplant group who were MRD positive following induction therapy, 9 (52.9%) of these patients were MRD negative 100 days after HSCT. The other 8 (47.1%) patients remained in CR with MRD. Details regarding MRD analysis are displayed in Figure 1.

### 3.2 Survival analysis

**Figure 2:**
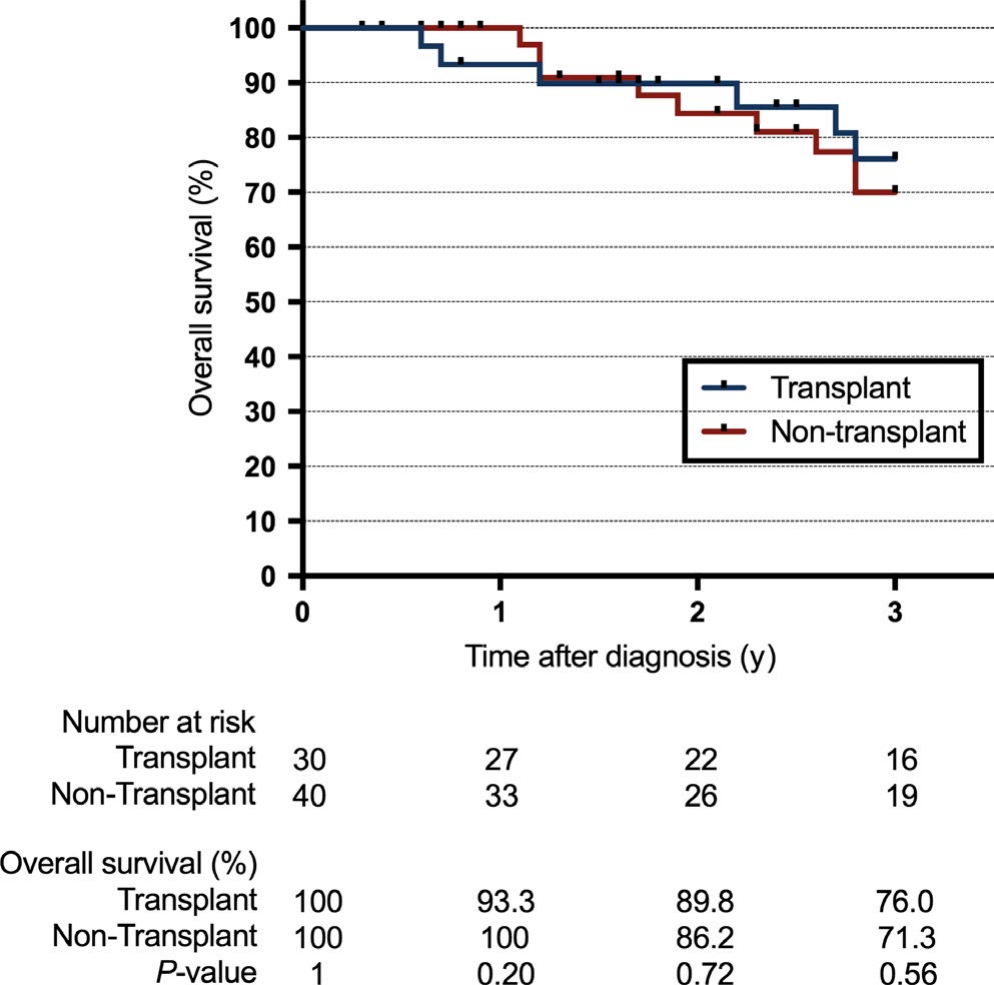
Overall survival since time of diagnosis

**Figure 3:**
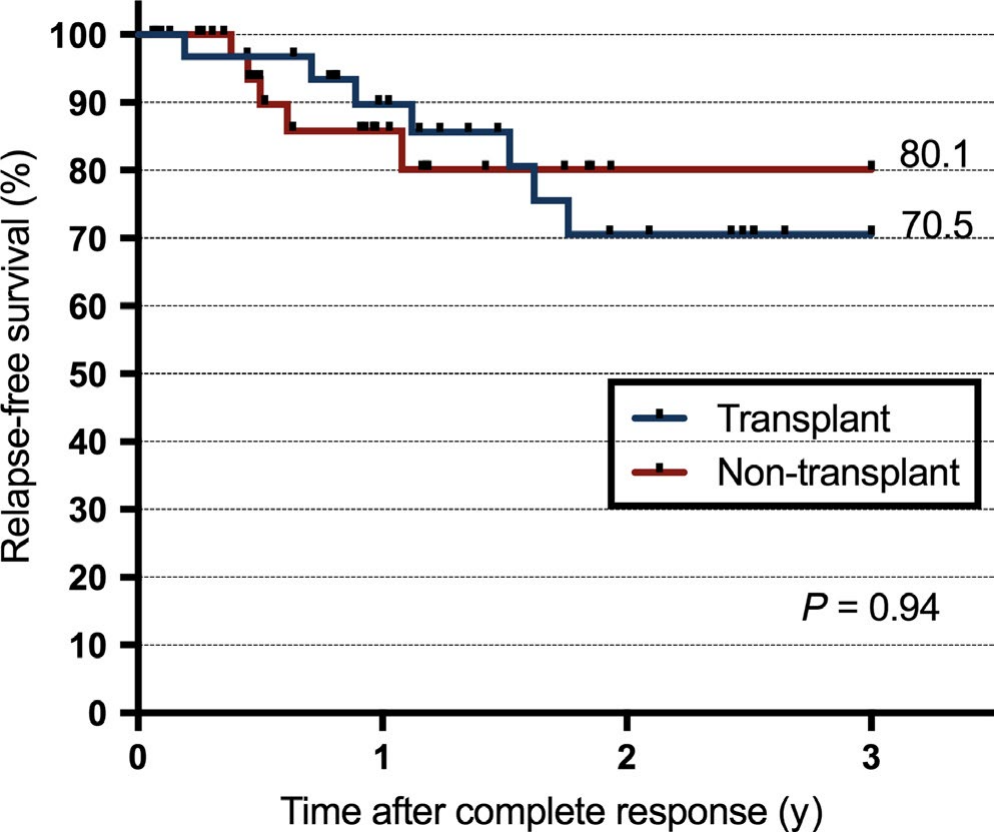
Relapse‐free survival since first complete response

The OS rates for all patients in the transplant and nontransplant groups were calculated at the 1‐, 2‐, and 3‐year marks. The 1‐year OS was 93.3% versus 100% (*P* = 0.20), 2‐year OS was 89.8% versus 86.2% (*P* = 0.72), and 3‐year OS was 76% versus 71.3% (*P* = 0.56) in the allogeneic HSCT group compared to the nontransplant group, respectively (Figure 2). Similar to the rates of OS, the 3‐year RFS rate also proved to be comparable at 70.5% in the allogeneic HSCT group and 80.1% in the nontransplant group (*P* = 0.94, Figure 3).

Subgroup analyses were performed for patients with the poor prognostic factors of a white blood count (WBC) >30 × 10^9^/L or age >35 years at time of diagnosis. A total of 8 (26.7%) patients in the transplant group and 12 (30%) patients in the nontransplant group had a WBC >30 × 10^9^/L when diagnosed. When comparing rates of 1‐year, 2‐year, and 3‐year OS as well 3‐year RFS between transplant versus nontransplant patients in this group, there was also no significant difference (*P* = 1 for 1‐year OS, *P* = 0.9 for 2‐year and 3‐year OS, *P* = 1 for 3‐year RFS). In addition, 21 (70%) patients in the transplant group and 29 (72.5%) patients in the nontransplant group were diagnosed with Ph+ ALL at an age > 35 years. Again, the rates of OS and RFS were not significantly different when analyzing transplant versus nontransplant patients in this older age group (*P* = 1 for 1‐year OS, *P* = 0.11 for 2‐year OS, *P* = 0.58 for 3‐year OS, *P* = 0.51 for 3‐year RFS).

Further analysis was completed for patients with confirmed MRD positive or MRD negative disease following induction therapy. In patients with MRD positive disease, there was no significant difference between the rates of OS up to 3‐years after diagnosis as well as rates of 3‐year RFS between the transplant and nontransplant groups (*P* = 1 for 1‐year and 2‐year OS, *P* = 0.25 for 3‐year OS, *P* = 0.47 for 3‐year RFS). Likewise, these outcomes were also similar when measured in the MRD negative population as well (*P* = 1 for all OS and 3‐year RFS).

Finally, patients were stratified by the type of chemotherapy that they received during the study: either the USC ALL regimen or a different regimen (hyper‐CVAD or the BFM‐like protocol). When analyzing the rates of OS between these 2 groups, there was a trend toward a survival advantage in the USC ALL regimen group with a 1‐year OS of 97.9% versus 95.7% (*P* = 1), 2‐year OS of 90.9% versus 83% (*P* = 0.44), and 3‐year OS of 78.5% versus 62.3% (*P* = 0.22) in those who received the USC ALL regimen versus those who received another type of chemotherapy, respectively.

## 4 DISCUSSION

In modern times, allogeneic HSCT continues to be highly utilized in the adult Ph+ ALL population. This can be attributed to prior studies demonstrating OS benefit of allogeneic HSCT compared to only chemotherapy in both the pre and post‐imatinib eras.^20^, ^21^ An updated analysis of the phase II GRAAPH‐2003 trial is one such example in which Ph+ ALL patients treated with chemotherapy plus imatinib were found to have significantly superior 4‐year OS rates following HSCT compared to maintenance imatinib alone (76%‐80% vs 33%).^22^ Nonetheless, the data comparing HSCT against combination chemotherapy with newer generation TKIs remains sparse.

Our study reports the real life experience of managing adult patients with Ph+ ALL using dasatinib as the primary TKI. We found no significant difference in OS or RFS up to the 3‐year time point between those who received chemotherapy plus dasatinib followed by allogeneic HSCT compared to those who received only chemotherapy plus dasatinib alone. This finding was in contrast to prior results demonstrating benefit from HSCT in which imatinib was the primary TKI used rather than newer generation agents. As the mechanism of dasatinib includes a greater range of kinase inhibition, including those that are involved in alternative signaling for imatinib‐resistant ALL, this could result in more potent and durable disease suppression. Additionally, the greater penetration of the blood brain barrier of dasatinib compared to imatinib is another notable benefit that could have contributed to the OS and RFS rates in this study. Patients in the nontransplant group were also continued on dasatinib indefinitely until disease progression, an approach supported by a previous phase II study demonstrating long‐term remission after receiving hyper‐CVAD plus dasatinib followed by continued dasatinib maintenance therapy.^23^

In the adult Ph+ ALL population, poor prognostic factors have historically included a higher initial WBC (>30 × 10^9^/L) or age >35 years at the time of diagnosis.^24^, ^25^ Our subgroup analyses of patients with these factors also showed no significant difference in OS as well as 3‐year RFS between transplant and nontransplant patients, despite previous reports demonstrating a survival benefit from HSCT in those with significant leukocytosis.^26^ The difference in patient therapy again includes the use of dasatinib in our study compared to imatinib in prior analyses, indicating a potential lack of benefit of HSCT in these poor prognostic subgroups when a newer generation TKI is utilized.

MRD status is another factor determined to have strong prognostic implications in ALL; in particular, MRD positivity is an independent predictor of disease relapse and is also more prevalent among the Ph+ population.^27^, ^28^ However, the results of our study again showed no difference in up to 3‐year OS or 3‐year RFS when comparing transplant versus nontransplant patients in both the MRD positive and MRD negative subgroups. Of note, transplant did demonstrate a notable benefit in the conversion of MRD positive to MRD negative disease in this study. As demonstrated by bone marrow biopsies performed 100 days after HSCT, 9 (52.9%) patients in CR with MRD prior to transplant were found to be in CR without MRD afterward. Though no direct comparison was made against the chemotherapy plus dasatinib group for this finding, the conversion to MRD negative status in the majority of HSCT patients is an important conclusion when considering future candidates for transplant.

In looking at prior studies, one multicenter trial also investigated the outcomes of adult Ph+ ALL patients who had undergone treatment with chemotherapy plus dasatinib.^29^ Similarities with our study include the comparison of patients who received chemotherapy plus dasatinib followed by allogeneic HSCT against those who received chemotherapy plus dasatinib alone. Likewise, all transplanted patients in the multicenter trial underwent HSCT while in CR1 with dasatinib subsequently started around day 100. In contrast to our results, this study found that patients who underwent transplant had superior RFS and OS rates compared to nontransplant patients during landmark analysis conducted 175 days after CR Notable differences from our study design include the chemotherapy regimens that were provided to patients; while all patients in the multicenter trial received hyper‐CVAD, several different regimens were used in our study including the USC ALL regimen and BFM‐like protocol in addition to hyper‐CVAD. As the USC ALL regimen was the most frequently utilized regimen, given to 67.1% of patients of our study, this may have contributed to our differing results from the multicenter trial. An analysis conducted in 2014 of patients who were treated with the USC ALL regimen demonstrated a CR in 96% of patients, almost all within 4 weeks of therapy.^19^ In addition, the 7‐year disease‐free survival (DFS) and OS rates were found to be 58% and 51%, respectively, indicating durable survival benefit from the regimen. These positive results previously shown from the USC ALL regimen suggest that it may be a particularly effective form of chemotherapy for ALL patients. Furthermore, stratifying patients by chemotherapy regimen in our study also demonstrated a trend toward a survival advantage in those who received the USC ALL regimen when analyzing rates of OS up to the 3‐year mark. While these results did not meet statistical significance, the trend toward an OS advantage suggests that a significant survival benefit may be demonstrated in future studies with larger patient populations and longer durations of follow‐up. Consequently, the use of the USC ALL regimen in addition to dasatinib in the majority of our patients is a notable aspect of our study, which distinguishes it from similar previous reports.

Along with dasatinib, other newer generation TKIs have been shown to have promising activity in Ph+ ALL. For example, ponatinib in combination with hyper‐CVAD was previously examined in a phase II prospective trial.^30^ Of the 32 patients with Ph+ ALL included in the study, a complete cytogenetic response was achieved in 32 (100%) patients. Flow cytometry also revealed that 35 (95%) patients had no MRD after a median of 3 weeks of therapy. A later study compared ponatinib plus hyper‐CVAD against dasatinib plus hyper‐CVAD as frontline therapy in adult Ph+ ALL, showing significantly higher 3‐year OS and 3‐year EFS rates in the former.^31^ Given the positive results shown from ponatinib thus far, future studies could be conducted to directly compare chemotherapy plus ponatinib against HSCT in order to determine whether there are similar, or even superior, outcomes.

There were several limitations to this study. The small number of patients in both the transplant and nontransplant groups precluded any definitive statements regarding the role of chemotherapy plus dasatinib over HSCT in Ph+ ALL. Furthermore, the relatively short follow‐up time was another drawback that limited what conclusions could be made from this study. Selection bias was another shortcoming as patients who underwent allogeneic HSCT were those who had donor availability, specific insurance coverage, and a caregiver at home.

To conclude, allogeneic HSCT has historically been viewed as a favorable therapeutic option in adult Ph+ ALL patients. However, its benefit in the era of newer generation TKIs remains unclear. In certain high‐risk patients, such as those with relapsed or refractory disease, transplant remains a reasonable treatment modality. There is also notable benefit amongst patients who do not become MRD negative early in their disease courses, as transplant was shown in this study to be associated with the eradication of MRD in the majority of HSCT patients. Our data suggest, however, that chemotherapy plus dasatinib alone may lead to comparable outcomes as chemotherapy plus dasatinib followed by allogeneic HSCT in a subset of adult Ph+ ALL patients in CR1. Given the retrospective nature of this study, further investigation is warranted to confirm these findings through larger prospective studies and identify which specific group of patients should be managed without undergoing transplant.
